# Fracture Analysis of MWCNT/Epoxy Nanocomposite Film Deposited on Aluminum Substrate

**DOI:** 10.3390/ma10040408

**Published:** 2017-04-13

**Authors:** Shiuh-Chuan Her, Pao-Chu Chien

**Affiliations:** Department of Mechanical Engineering, Yuan Ze University, Chung-Li 320, Taiwan; s1005043@mail.yzu.edu.tw

**Keywords:** multi-walled carbon nanotube, nanocomposite film, strain energy release rate, fracture toughness

## Abstract

Multi-walled carbon nanotube (MWCNT) reinforced epoxy films were deposited on an aluminum substrate by a hot-pressing process. Three-point bending tests were performed to determine the Young’s modulus of MWCNT reinforced nanocomposite films. Compared to the neat epoxy film, nanocomposite film with 1 wt % of MWCNT exhibits an increase of 21% in the Young’s modulus. Four-point-bending tests were conducted to investigate the fracture toughness of the MWCNT/epoxy nanocomposite film deposited on an aluminum substrate with interfacial cracks. Based on the Euler-Bernoulli beam theory, the strain energy in a film/substrate composite beam is derived. The difference of strain energy before and after the propagation of the interfacial crack are calculated, leading to the determination of the strain energy release rate. Experimental test results show that the fracture toughness of the nanocomposite film deposited on the aluminum substrate increases with the increase in the MWCNT content.

## 1. Introduction

Carbon nanotubes (CNTs) have been utilized as nanoscale reinforcements due to their high elastic modulus, strength, and flexibility, combined with their low density and negligible thermal expansion [1,2,3,4]. Nanoscale fillers with larger surface areas in composites could lead to very large degrees of stress transfer and energy dissipation, resulting in a very high strength and toughness of nanocomposites [5]. An addition of small amounts of CNTs to polymer matrices yielded a significant improvement of mechanical properties [6,7,8]. Furthermore, the excellent electrical and optical properties of CNTs can be used to develop multifunctional products [9]. It is well known that Van der Waals interactions between small-diameter nanotubes result in the aggregates of nanotube ropes. This interlacing of carbon nanotubes is a significant barrier toward the transfer of load to the nanotube via shear stresses at the nanotube/matrix interface. As a result, the effective stiffness of multi-walled nanotubes in a polymer matrix is reduced [10]. Surface functionalization of CNTs is effective in improving dispersion and their adhesion in epoxy matrices. Sun et al. [11] investigated the effects of CNT dispersion and surface treatment on the mechanical properties of CNT-reinforced epoxy composites. They found that incorporation of CNTs grafted with polyamidoamine generation-0 (PAMAM-0) can improve the epoxy Young’s modulus and tensile strength by 18% and 16%, respectively. These improvements are not as significant as expected due to the significant curvature of CNTs in epoxy and the side reaction between the epoxy and the functionalization agent. Guadagno et al. [12] reported that nanotube functionalization with –COOH has a strong influence on the electrical properties and results in a remarkable reduction of the conductance of the composite. Yang et al. [13] grafted triethylenetetramine (TETA) on the MWCNT surface. The existence of TETA on MWCNT can improve the MWCNT–epoxy interfacial interaction and, thus, effectively enhance the mechanical properties of MWCNT/epoxy composites. They found that the impact strength, bending strength, and bending modulus of the 0.6 wt % content TETA-MWCNT/epoxy composite increased by 84%, 29%, and 22%, respectively, compared with those of the pure epoxy matrix.

Various dispersion methods (e.g., stirring, extrusion, kneading, etc.) for the distribution of CNTs in polymers have been used. Thostenson and Chou [14] proposed a scalable calendering approach for the dispersion of CVD-grown multi-walled carbon nanotubes through intense shear mixing. The as-processed nanocomposites exhibited significantly enhanced fracture toughness at low nanotube concentrations. The thermal conductivity increased linearly with nanotube concentration to a maximum increase of 60% at 5 wt % of carbon nanotubes. Hsieh et al. [15] investigated the fracture energy and fatigue performance of an anhydride-cured epoxy modified by MWCNT. They reported that the fracture energy increased, from 133 to 223 $J/m^{2}$ with the addition of 0.5 wt % of nanotubes. The addition of the CNTs also resulted in the increase in the fatigue performance. The threshold strain-energy release rate increased from 24 $J/m^{2}$ for the unmodified material to 73 $J/m^{2}$ for the epoxy with 0.5 wt % CNTs. Yu et al. [16] studied the mode-I fracture toughness and fatigue life of multi-walled carbon nanotube-reinforced epoxy-matrix composites. Experimental results showed that the average fracture toughness of 1 wt %- and 3 wt %-MWCNT/epoxy composites was 1.29 and 1.62 times of that of pure epoxy, respectively. The 0.5 wt %-MWCNT/epoxy composites’ fatigue lives were 10.5 and 9.3 times of the average fatigue life of neat epoxy. Tang et al. [17] developed a novel method to investigate the failure modes of MWCNT in epoxy matrix by the use of SEM. They proposed three types of failure modes, i.e., pullout, immediate fracture, and sliding-fracture, to explain the fractographs. Tang et al. [18] investigated the fracture mechanisms and reinforcing effects of ozone-treated multi-walled carbon nanotubes in epoxy matrix. They found that the MWCNT failure modes transferred from pullout to sliding fracture after ozone functionalization. This could favor energy consumption and, thus, contribute more to fracture toughness improvements. Domun et al. [19] reviewed the effects of the addition of different nanoparticles, such as single-walled CNT (SWCNT), double-walled CNT (DWCNT), multi-walled CNT (MWCNT), graphene, nanoclay and nanosilica, on fracture toughness, strength, and stiffness of the epoxy matrix. The review shows that, depending on the type of nanoparticles, the integration of the nanoparticles has a substantial effect on mode I and mode II fracture toughness, strength, and stiffness. Sue and White [20] prepared Epoxy/Polyamide-12/MWCNT ternary composites using ultrasonication and solvent evaporation. Ternary composites with epoxy/PA/MWCNT exhibit significantly enhanced fracture toughness due to the MWCNT network acting to bridge between PA particles and increase participation in the fracture process.

Carbon-based nanocomposite films have received great attention due to their excellent electrical, mechanical, thermal, and optical properties. Depending on the film composition and preparation method, a variety of applications have been used for carbon nanocomposite films. Li et al. [21] prepared MWCNT/polyacrylate composites film applied on a building interior wall for electromagnetic interference (EMI) shielding applications. Ganash [22] investigated the corrosion behavior of polypyrrole and polypyrrole/carbon nanotube nanocomposite films deposited on 304 stainless steel surfaces using the electrochemical synthesis method. Conductive polymer composites containing MWCNT exhibit a variety of sensing performance when subjected to external strain. Sanli et al. [23] investigated the piezoresistive properties of flexible, strain sensitive, multi-walled carbon nanotubes (MWCNT)/epoxy composite film using electrochemical impedance spectroscopy. They found that higher sensitivity is achieved in comparison with traditional strain gauges. Cao et al. [24] studied the strain sensing behavior of multi-walled carbon nanotubes (MWCNT)/epoxy (EP) conductive composites subjected to cyclic deformation. They reported that the change of the resistance increased in a linear fashion and then began to decrease at a critical strain, which was remarkably different from the thermoplastic conductive composites, only with a monotonic increase. These behaviors were attributed to the competition of network destruction and reconstruction during the cyclic deformation. Pham et al. [25] studied the strain-dependent resistivity of the poly (methyl methacrylate)/MWCNT composites. Wang et al. [26] proposed a high temperature strain sensor based on polyimide/single-wall carbon nanotube (SWCNT) composites. Several strain sensors with different SWCNT contents ranging from 0.07 to 2.0 wt % were investigated. They found that the optimum CNT concentration for the sensor is 1.4 wt %.

Review of the studies mentioned above shows that most of the existing works investigate the mechanical properties of MWCNT-reinforced composite in bulk state. Very few efforts have been made to address the mechanical behavior of MWCNT-reinforced nanocomposite film deposited on a substrate. As the nanocomposite film is deposited on the substrate, delamination is the most common defect in the film/substrate composite structure. Interfacial cracks between the film and substrate as a result of delamination can induce significantly high stress at the crack tip leading to the failure of the film/substrate composite structure. Thus, it is essential to evaluate the interfacial fracture toughness which can be used to characterize the capability of the film/substrate composite in resistance to the crack growth. To the best of our knowledge, there is no report dealing with the fracture toughness of the nanocomposite film deposited on a substrate with an interfacial crack. In addition, determination of the Young’s modulus for a film deposited on the substrate is quite different from a free-standing film. Two novel technologies based on the three-point bending and four-point bending tests were proposed to determine the Young’s modulus and critical strain energy release rate of the nanocomposite film deposited on a substrate, respectively. In this work, multi-walled carbon nanotube (MWCNT)-reinforced epoxy thin films were prepared on an aluminum substrate through the sonication and hot-pressing processes. The aim of this study is to investigate the effect of MWCNT content on the Young’s modulus and fracture toughness of the nanocomposite film deposited on the aluminum substrate. Young’s modulus can be used to evaluate the capability of a material in resistance to deformation. The critical strain energy release rate is an intrinsic property of a material which characterizes the ability of a material to dissipate the deformation energy without propagation of a crack. Thus, the critical strain energy release rate can be used to judge the material toughness.

## 2. Materials and Experimental Processes

### 2.1. Fabrication of Nanocomposite Films

**Figure 1:**
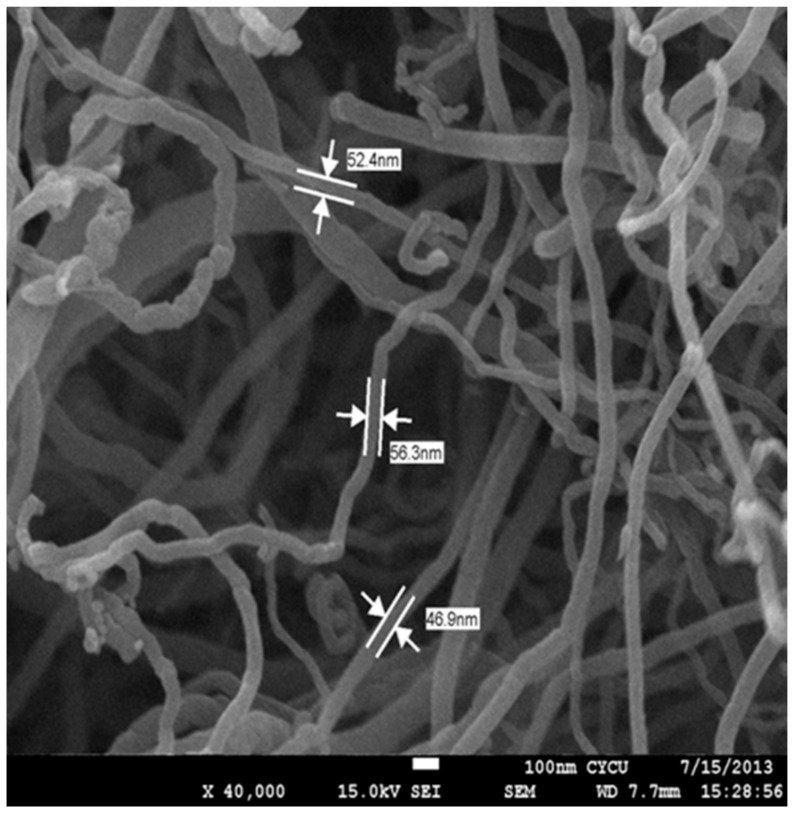
SEM image of the multi-walled carbon nanotubes.

The multi-walled carbon nanotubes produced via the chemical vapor deposition method were purchased from Uchess Co. (New Taipei, Taiwan) and purified to >95%. The MWCNT had a diameter ranging from 40 nm to 60 nm, and a length ranging from 5 μm to 15 μm. Figure 1 shows the image of the MWCNT using a field emission scanning electron microscope (JSM 7600F, Jeol, Tokyo, Japan). The matrix used was part A: epoxy Mungo 4200A and part B: hardener 4200B, both purchased from Glad Co. (New Taipei, Taiwan). Ultrasonic cavitation is an efficient method to disperse carbon nanotubes into epoxy resin when CNT weight fractions are lower than 1.0 wt %. However, high ultrasonic power or long duration may damage the CNT surface. In this study, multi-walled carbon nanotubes were incorporated into epoxy matrix through a high-intensity ultrasonic bath process. A series of experimental tests were conducted to determine the optimal ultrasonic power and duration that can effectively achieve good dispersion without damaging the CNT surface. The optimal ultrasonic power and duration were 120 W and 3 h, respectively. The dispersion of MWCNTs in the epoxy matrix were examined using SEM.

**Figure 2:**
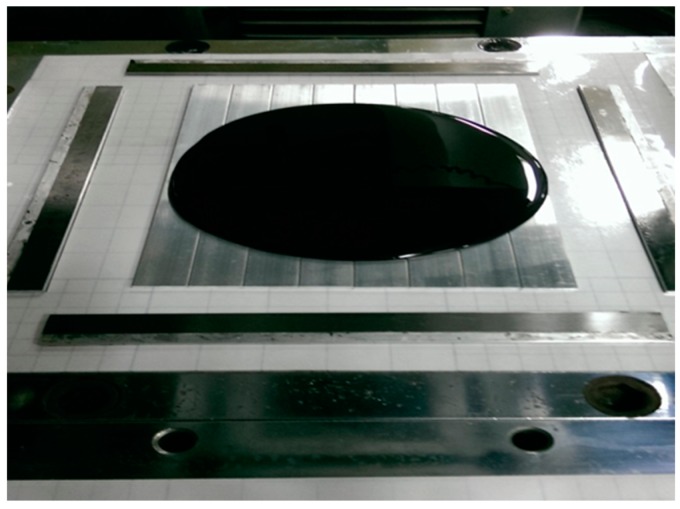
Nanocomposite suspension on the hot pressing mold.

**Figure 3:**
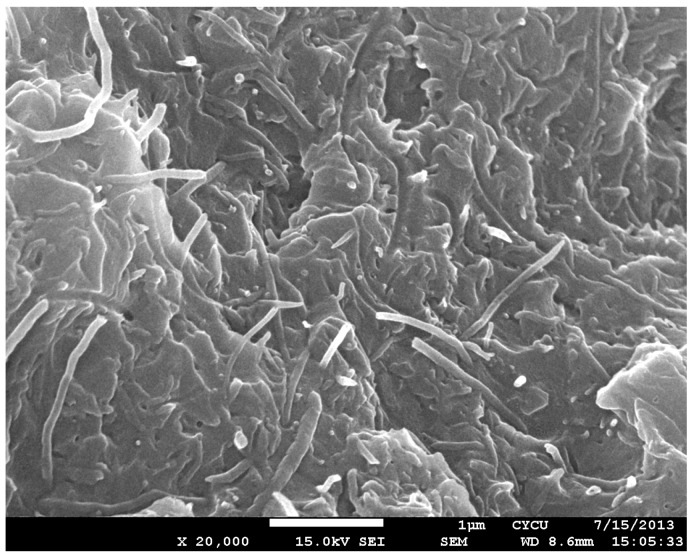
SEM image of nanocomposite with 1.0 wt % of MWCNT.

To prepare MWCNT/epoxy nanocomposite, MWCNT powder was directly added into a liquid epoxy, and the solution was sonicated for 3 h at temperature of 40 °C to separate the aggregation of MWCNTs and achieve a good dispersion. Then, the epoxy hardener was mixed into the MWCNT/epoxy solution at a weight ratio of 1:2, and softly stirred for about 5 min. After that, the solution was placed in a vacuum chamber for about 30 min to remove the bubbles induced from the stirring. Finally, the nanocomposite suspension was poured onto a clean aluminum substrate, which is placed on a mold as shown in Figure 2. The mold was supported and covered by steel plates and placed on the hot-press machine at temperature of 40 °C and under a pressure of $400\;N/{\mathrm{cm}}^{2}$. The thickness of the nanocomposite film on the substrate was controlled by a spacer. Prior to pouring the nanocomposite solution, the substrate was cleaned in a soap solution, and submerged in an acetone solution for 10 min after rising with distilled water. Then the substrate was dried with hot air before pouring. A nanocomposite film with a thickness of 200 μm was obtained after one day of curing in the hot-pressing mold. Four different loadings of MWCNT (0.3, 0.5, 0.8, and 1.0 wt %) were prepared to investigate the effect of the MWCNT content on the mechanical properties of the nanocomposite film. Samples without the MWCNT addition were also fabricated for comparison. Figure 3 depicted the SEM image of the nanocomposite with 1.0 wt % of MWCNT. This shows good dispersion and good bonding between the MWCNT and epoxy.

### 2.2. Three-Point Bending Test

The Young’s modulus of nanocomposite film deposited on a substrate was determined by a three-point bending test. In a bending test, the deflection of the film/substrate composite beam is dependent on the bending stiffness of the composite beam, which is related to the elastic moduli of both the film and substrate. Thus, the Young’s modulus of the film can be deduced by comparing the deflections between the substrate and film/substrate specimens. In this work, a simple relation derived by Bao et al. [27] was adopted to determine the elastic modulus of the film from the three-point bending test. Based on beam theory, the deflection of a beam subjected to a three-point bending test can be written as:

(1) $$v=\frac{{\mathrm{PL}}^{3}}{48\mathrm{EI}}$$

where P, L, E, and I represent the applied load, span, Young’s modulus, and the moment of inertia of the beam, respectively. For the film/substrate composite beam, the position of the neutral axis $y_{c}$ and bending stiffness $\bar{E}\;\bar{I}$ are as follows:

(2) $$y_{c}=\frac{E_{f}{\;h}_{f}\left(h_{f}+2h_{s}\right)+E_{s}{\;h}_{s}^{2}}{2\left(E_{f}{\;h}_{f}+E_{s\;}h_{s}\right)}$$

(3) $$\bar{E}\;\bar{I}==\frac{b}{12}\left[E_{f}\;h_{f}^{3}+E_{s}\;h_{s}^{3}+3E_{f}E_{s}h_{f}h_{s}\frac{{\left(h_{f}+h_{s}\right)}^{2}}{E_{f}h_{f}+E_{s}\;h_{s}}\right]$$

where $E_{f}$ and $E_{s}$ denote the Young’s modulus of the film and substrate, respectively; $h_{f}$ and $h_{s}$ are the thickness of the film and substrate and b is the width of the composite beam, respectively.

For the beam with pure substrate, the slope of the load-deflection curve derived from Equation (1) yields:

(4) $${\left(\frac{\mathrm{dP}}{\mathrm{dv}}\right)}_{s}=\frac{48}{L^{3}}\frac{E_{s}{\;b\;h}_{s}^{3}}{12}$$

For the composite beam consisting of film and substrate, the slope of the load-deflection curve is:

(5) $${\left(\frac{\mathrm{dP}}{\mathrm{dv}}\right)}_{c}=\frac{48}{L^{3}}\bar{E}\;\bar{I}$$

From Equations (3)–(5), the slope ratio γ between the composite beam and substrate can be expressed as:

(6) γ=(dPdv)c(dPdv)s=[1+EfEs(hfhs)3+3Ef hf (hfhs+1)2Ef hf+Es hs

Equation (6) is rewritten as:

(7a) $$c_{1}{\;\beta }^{2}+c_{2}\;\beta +c_{3}=0$$

(7b) $$\beta =\frac{E_{f}}{E_{s}}$$

(7c) $$c_{1}={\left(\frac{h_{f}}{h_{s}}\right)}^{4};\;c_{3}=1-\gamma$$

(7d) $$c_{2}=\frac{h_{f}}{h_{s}}[{\left(\frac{h_{f}}{h_{s}}\right)}^{2}+3\left(1+\frac{h_{f}}{h_{s}}{)}^{2}+\left(1-\gamma \right)\right]$$

The slope ratio γ is determined by the three-point bending test. Thus, β can be obtained from Equation (7a) as follows:

(8) $$\beta =\frac{-c_{2}+\sqrt{c_{2}^{2}-4c_{1\;}c_{3}}}{2c_{1}}$$

Substituting Equation (8) into Equation (7b) leads to the Young’s modulus of the film as follows:

(9) $$E_{f}={\beta \;E}_{s}=\frac{-c_{2}+\sqrt{c_{2}^{2}-4c_{1}{\;c}_{3}}}{2c_{1}}E_{s}$$

### 2.3. Four-Point Bending Test

**Figure 4:**
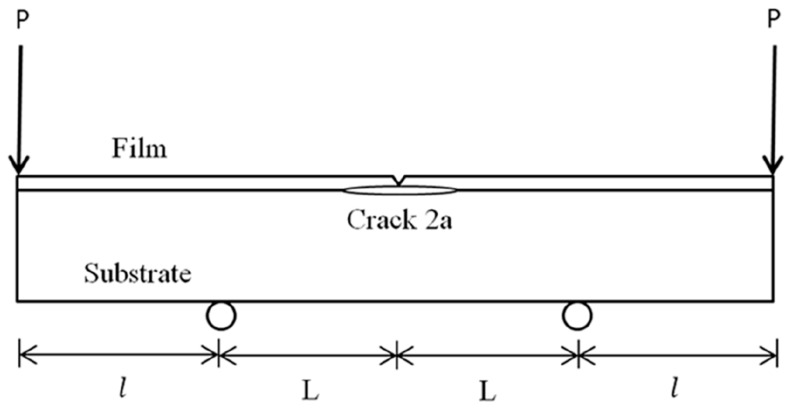
Four-point bending of the film/epoxy composite beam with an interfacial crack.

**Figure 5:**
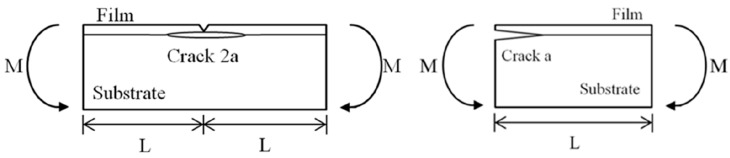
The pure bending moment in the middle region of the composite beam.

The fracture toughness of the nanocomposite film deposited on the substrate with an interfacial crack was determined by a four-point bending test. A central notch is cut through the thickness of the nanocomposite film and a symmetrical crack is placed along the interface with a crack length of 2a as shown in Figure 4. The Young’s modulus and thickness of the nanocomposite film are $E_{f}$ and $h_{f}$, respectively, $E_{s}$ and $h_{s}$ are associated with the substrate. The specimen is subjected to the four-point bending test. The interfacial crack located between the two inner loading points is under a constant moment condition. The strain energy release rate exhibits steady-state characteristics when the crack length is significantly exceeded to the thickness of the nanocomposite film. The specimen in the steady-state region is subjected to a pure bending moment M=P×l as shown in Figure 5. As the length of the crack extends from 2a to 2a+2δa, the difference of the strain energy in the specimen before and after the crack propagation is simply the difference of the strain energy stored in the center and at the free end.

(10) $$\delta W=\int_{0}^{\delta a}\frac{M^{2}}{2E_{s}I_{s}}dx-\int_{0}^{\delta a}\frac{M^{2}}{2\bar{E}\bar{I}}dx$$

M=P×l

The strain energy release rate of nanocomposite film deposited on the substrate with interfacial crack is readily to be determined as follows:

$$G=\underset{\delta A\to 0}{\lim}\left|\frac{\delta W}{\delta A}\right|=\underset{\delta a\to 0}{\lim}\left|\frac{\delta W}{b\delta a}\right|$$

(11) $$G=\frac{6{\left(Pl\right)}^{2}}{b^{2}}\left[\frac{1}{E_{s}h_{s}^{3}}-\frac{E_{f}h_{f}+E_{s}h_{s}}{\left(E_{f}h_{f}^{3}+E_{s}h_{s}^{3}\right)\left(E_{f}h_{f}+E_{s}h_{s}\right)+3E_{f}E_{s}h_{f}h_{s}{\left(h_{f}+h_{s}\right)}^{2}}\right]$$

## 3. Results and Discussions

In this section, a three-point bending test was employed to evaluate the Young’s modulus of the nanocomposite film, while four-point bending tests were conducted to determine the fracture toughness.

### 3.1. Young’s Modulus of the Nanocomposite Film

**Figure 6:**
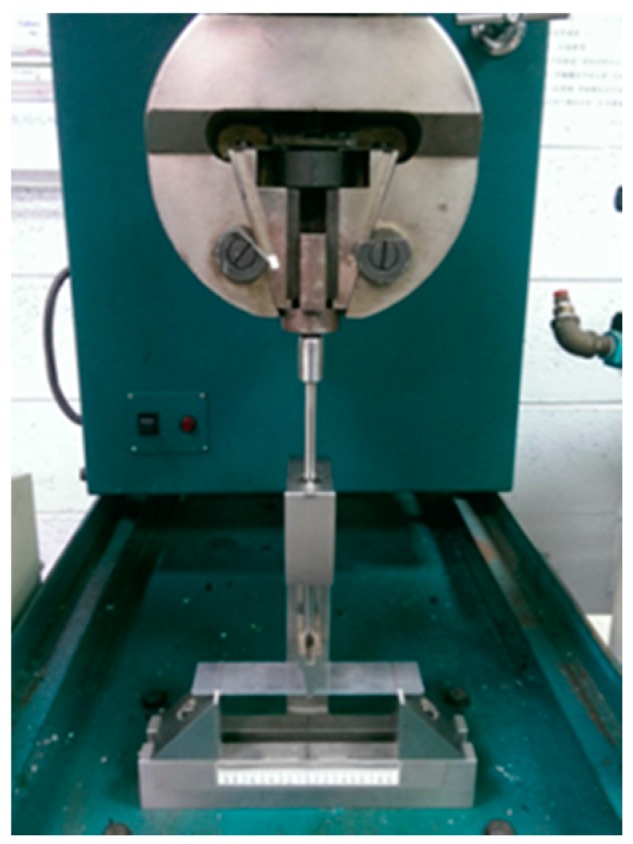
Experimental setup of the three-point bending test.

**Figure 7:**
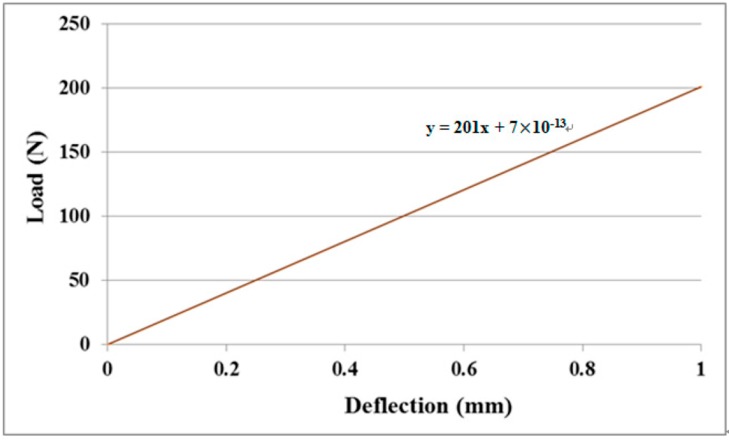
Load-deflection curve of aluminum substrate.

**Figure 8:**
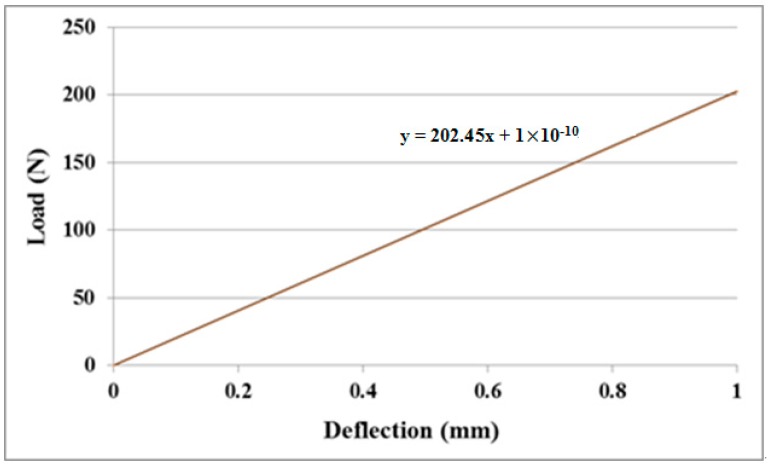
Load-deflection curve of film/substrate composite beam with MWCNT 0.5 wt %.

MWCNT/epoxy nanocomposite films with four different contents of MWCNT (0.3 wt %, 0.5 wt %, 0.8 wt %, and 1.0 wt %) were prepared and deposited on the aluminum substrate to form the film/substrate composite beam. The Young’s modulus, length, width, and thickness of the aluminum substrate are 70 GPa, 125 mm, 25 mm, and 3 mm, respectively. The thickness of the nanocomposite film is 0.2 mm. The experimental setup of the three-point bending test is shown in Figure 6. Four tests were performed for each sample and the average of these tests was presented in this work. A load is applied on the middle of the specimen. The load (p) and deflection (v) of the three-point bending test are recorded by the universal testing machine. The load-deflection curves for the aluminum substrate and film/substrate composite beam with a MWCNT of 0.5 wt % are plotted in Figure 7 and Figure 8, respectively. It can be observed that both the load-deflection curves are linear. The slopes of the load-deflection curves for aluminum substrate ${\left(\frac{dp}{dv}\right)}_{s}$ and composite beam ${\left(\frac{dp}{dv}\right)}_{c}$ are easily extracted from these curves. Therefore, the slope ratio $\gamma ={\left(\frac{dp}{dv}\right)}_{c}/{\left(\frac{dp}{dv}\right)}_{s}$ between the composite beam and the aluminum substrate can be determined. Substituting the film thickness $h_{f}$, substrate thickness $h_{s}$, and slope ratio γ into Equations (7) and (9) leads to the determination of the Young’s modulus of the nanocomposite film. Table 1 lists the Young’s moduli of the nanocomposite films with MWCNT contents ranging from 0.3 wt % to 1.0 wt %. This shows that the Young’s modulus of the nanocomposite film is increasing with the increase of the MWCNT content. The Young’s modulus of the nanocomposite film with 1.0 wt % of MWCNT is increased by 21% when compared with neat epoxy film. We also conducted the tensile test according to the ASTM D638 standard to determine the Young’s modulus of the neat epoxy. The result of the Young’s modulus from the tensile test for neat epoxy is 1.99 GPa, which agrees well with the result of 1.90 GPa from the three-point bending test, with a difference of less than 5%. This demonstrates that the nanocomposite film is firmly adhered to the Al substrate, resulting in a good strain transfer from the substrate to the film. The SEM image of the nanocomposite with 1.0 wt % of MWCNT shown in Figure 3 illustrates good dispersion and bonding between the MWCNT and epoxy. The bonds between the nanotubes and the polymer enable a stress transfer and carrying of tensile loads which leads to the improvement of the Young’s modulus. In addition, the dispersion of the MWCNT that restricts the mobility of polymer chains under loading enhances the modulus and strength.

### 3.2. Strain Energy Release Rate

**Figure 9:**
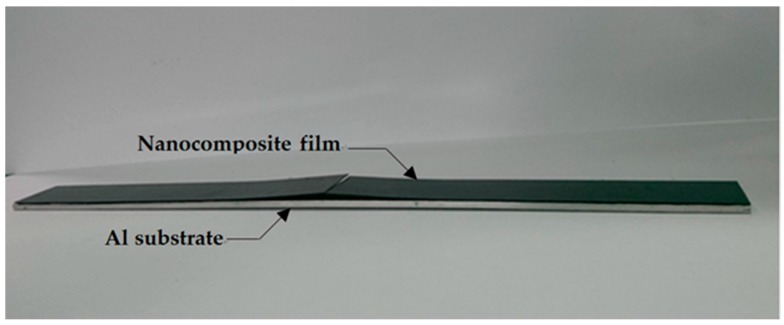
Film/substrate composite beam with an interfacial crack.

**Figure 10:**
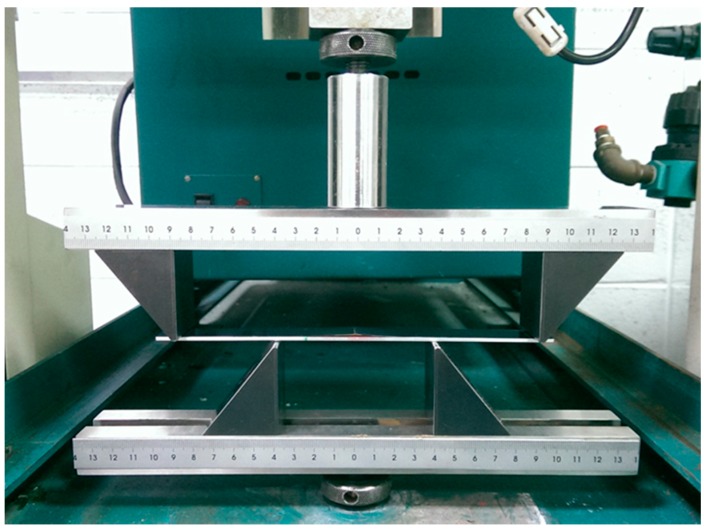
Experimental setup of the four-point bending test.

**Figure 11:**
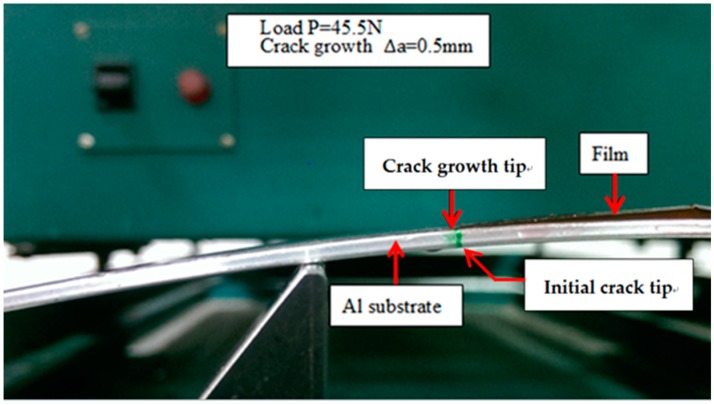
Interfacial crack starting to propagate at the critical load.

The strain energy release rate of the nanocomposite film deposited on the aluminum substrate with an interfacial crack was derived in Section 4. A four-point bending test was conducted to determine the fracture toughness of the film/substrate composite beam. The length, width, and thickness of the aluminum substrate are 200 mm, 19 mm, and 2 mm, respectively. The thicknesses of the film and interfacial crack length are 0.2 mm and 50 mm, respectively. Figure 9 shows the film/substrate composite beam with an interfacial crack. The experimental setup of the four-point bending test is shown in Figure 10. The middle region of the composite beam is subjected to a pure bending moment of M=P×l where P is the load and l=50 mm. The load is gradually increasing to the critical load $P_{cr}$ The interfacial crack propagation process is shown in Figure 11 where the crack tip is marked in green. The interfacial crack starts to propagate as the load increases to the critical load $P_{cr}$. The strain energy release rate can be determined by substituting the critical load $P_{cr}$ into Equation (11). Three test specimens were conducted for each sample and the average of these tests was presented in this work. The strain energy release rates of the nanocomposite film with various MWCNT contents deposited on the aluminum substrate are listed in Table 2. This shows that the strain energy release rate is increasing with the increase of the MWCNT content. The strain energy release rate of the nanocomposite film with 1.0 wt % of MWCNT is increased by 55% when compared with neat epoxy film. The increase of the fracture energy of the MWCNT reinforced epoxies was predicted by considering the contributions of the toughening mechanisms, which include nanotube debonding, nanotube pull-out, and plastic void growth of the epoxy matrix. Pull-out requires the CNTs to debond from the matrix, and there is likely energy dissipation from interfacial debonding. Once debonding has occurred, the constraint on the epoxy is relieved, and so plastic void growth may also occur. Such mechanisms may, indeed, account for the observed increases in the critical strain energy release rate. Crack-bridging is another micro-mechanical mechanism that reduces the growth of nano-pores, as well as the propagation of cracks, and contributes positively to the increase in fracture toughness.

## 4. Conclusions

Nanocomposite films reinforced with MWCNT were successfully prepared and deposited on an aluminum substrate via the sonication and hot pressing processes. Based on beam theory, an analytical relationship among the moduli of the film, substrate, and film/substrate was presented, from which the Young’s modulus of the nanocomposite film can be determined by a three-point bending test. Experimental results show that the Young’s modulus of the nanocomposite increases with the increase of the MWCNT content. Four-point bending tests were conducted to investigate the fracture toughness of MWCNT/epoxy nanocomposite film deposited on the aluminum substrate with an interfacial crack. This showed that the strain energy release rate increased with the increase of the MWCNT content. The strain energy release rate of the nanocomposite film with 1.0 wt % of MWCNT is increased by 55% when compared with neat epoxy film. The addition of MWCNT into the epoxy matrix shows a significant improvement of the fracture toughness to the nanocomposite film.

## Figures and Tables

**Table 1:** Young’s modulus (GPa) of the nanocomposite film with various MWCNT contents.

Specimen MWCNT wt %	1	2	3	4	Average	Increase
0%	1.90	1.90	1.90	1.90	1.90 ± 0.0	
0.3%	2.08	2.08	2.08	2.08	2.08 ± 0.0	9.43%
0.5%	2.17	2.18	2.18	2.18	2.18 ± 0.01	14.48%
0.8%	2.25	2.25	2.25	2.25	2.25 ± 0.0	18.22%
1%	2.30	2.31	2.30	2.31	2.30 ± 0.01	21.2%

**Table 2:** Strain energy release rate of the nanocomposite film deposited on the aluminum substrate with various MWCNT contents.

MWCNT wt %	Specimen 1		Specimen 2		Specimen 3		Average Strain Energy Release Rate $G_{I}$ (J/m^2^)
	Critical Load $P_{cr}$ (N)	Strain Energy Release Rate $G_{I}$ (J/m^2^)	Critical Load $P_{cr}$ (N)	Strain Energy Release Rate $G_{I}$ (J/m^2^)	Critical Load $P_{cr}$ (N)	Strain Energy Release Rate $G_{I}$ (J/m^2^)	
0%	45.5	151.81	45.4	150.82	45.4	150.66	150.10 ± 0.72
0.3%	47.5	180.75	47.7	181.87	47.4	179.63	180.75 ± 1.12
0.5%	48.9	200.10	48.7	198.47	48.7	198.39	198.99 ± 1.11
0.8%	50	216.14	49.9	214.81	50.2	217.46	216.14 ± 1.33
1%	51.2	231.86	51.2	231.85	51.4	233.67	232.46 ± 1.21

## References

[1] Lourie O., Wagner H.D. (1998). Evaluation of Young’s modulus of carbon nanotubes by micro-Raman spectroscopy. J. Mater. Res..

[2] Chou T.W., Gao L., Thostenson E.T., Zhang Z., Byun J.H. (2010). An assessment of the science and technology of carbon nanotube-based fibers and composites. Compos. Sci. Technol..

[3] Lourie O., Wagner H.D. (1999). Evidence of stress transfer and formation of fracture clusters in carbon nanotube-based composites. Compos. Sci. Technol..

[4] Eitan A., Jiang K., Dukes D., Andrews R., Schadle L.S. (2003). Surface modification of multiwalled carbon nanotubes: Toward the tailoring of the interface in polymer composites. Chem. Mater..

[5] Lachman N., Wagner H.D. (2010). Correlation between interfacial molecular structure and mechanics in CNT/epoxy nano-composites. Compos. Part A.

[6] Frogley M.D., Ravich D., Wagner H.D. (2003). Mechanical properties of carbon nanoparticle-reinforced elastomers. Compos. Sci. Technol..

[7] Fidelus J.D., Wiesel E., Gojny F.H., Schulte K., Wagner H.D. (2005). Thermo-mechanical properties of randomly oriented carbon/epoxy nanocomposites. Compos. Part A.

[8] Hussain F., Hojjati M. (2006). Polymer–matrix nanocomposites, processing, manufacturing, and application: An overview. J. Compos. Mater..

[9] Wang Q.H., Setlur A.A., Lauerhaas J.M., Dai J.Y., Seelig E.W., Chang R.P.H. (1998). A nanotube-based field-emission flat panel display. Appl. Phys. Lett..

[10] Li C.Y., Chou T.W. (2003). Elastic moduli of multi-walled carbon nanotubes and the effect of van der Waals forces. Compos. Sci. Technol..

[11] Sun L., Warren G.L., O’Reilly J.Y., Everett W.N., Lee S.M., Davis D., Lagoudas D., Sue H.-J. (2008). Mechanical properties of surface-functionalized SWCNT/epoxy composites. Carbon.

[12] Guadagno L., De Vivo B., Di Bartolomeo A., Lamberti P., Sorrentino A., Tucci V., Vertuccio L., Vittoria V. (2011). Effect of functionalization on the thermo-mechanical and electrical behavior of multi-wall carbon nanotube/epoxy composites. Carbon.

[13] Yang K., Gu M., Guo Y., Pan X., Mu G. (2009). Effects of carbon nanotube functionalization on the mechanical and thermal properties of epoxy composites. Carbon.

[14] Thostenson E.T., Chou T.W. (2006). Processing-structure-multi-functional property relationship in carbon nanotube/epoxy composites. Carbon.

[15] Hsieh T.H., Kinloch A.J., Taylor A.C., Kinloch I.A. (2011). The effect of carbon nanotubes on the fracture toughness and fatigue performance of a thermosetting epoxy polymer. J. Mater. Sci..

[16] Yu N., Zhang Z.H., He S.Y. (2008). Fracture toughness and fatigue life of MWCNT/epoxy composites. Mater. Sci. Eng. A.

[17] Tang L.C., Zhang H., Wu X.P., Zhang Z. (2011). A novel failure analysis of multi-walled carbon nanotubes in epoxy matrix. Polymer.

[18] Tang L.C., Zhang H., Han J.H., Wu X.P., Zhang Z. (2011). Fracture mechanisms of epoxy filled with ozone functionalized multi-wall carbon nanotubes. Compos. Sci. Technol..

[19] Domun N., Hadavinia H., Zhang T., Sainsbury T., Liaghat G.H., Vahid S. (2015). Improving the Fracture Toughness and the Strength of Epoxy Using Nanomaterials—A Review of the Current Status. Nanoscale.

[20] White K.L., Sue H.-J. (2011). Electrical Conductivity and Fracture Behavior of Epoxy/Polyamide-12/Multiwalled Carbon Nanotube Composites. Polym. Eng. Sci..

[21] Li Y., Chen C., Zhang S., Ni Y., Huang J. (2008). Electrical conductivity and electromagnetic interference shielding characteristics of multiwalled carbon nanotube filled polyacrylate composite films. Appl. Surf. Sci..

[22] Ganash A.A. (2014). Electrochemical synthesis and corrosion behaviour of polypyrrole and polypyrrole/carbon nanotube nanocomposite films. J. Compos. Mater..

[23] Sanli A., Müller C., Kanoun O., Elibol C., Wagner M.F.-X. (2016). Piezoresistive characterization of multi-walled carbon nanotube-epoxy based flexible strain sensitive films by impedance spectroscopy. Compos. Sci. Technol..

[24] Cao X., Wei X., Li G., Hu C., Dai K., Guo J., Zheng G., Liu C., Shen C., Guo Z. (2017). Strain sensing behaviors of epoxy nanocomposites with carbon nanotubes under cyclic deformation. Polymer.

[25] Pham G.T., Park Y.B., Liang Z., Zhang C., Wang B. (2008). Processing and modeling of conductive thermoplastic/carbon nanotube films for strain sensing. Compos. Part B.

[26] Wang Y., Wang A.X., Wang Y., Chyu M.K., Wang Q.M. (2013). Fabrication and characterization of carbon nanotube–polyimide composite based high temperature flexible thin film piezoresistive strain sensor. Sens. Actuators A.

[27] Bao Y.W., Zhou Y.C., Bu X.X., Qiu Y. (2007). Evaluating elastic modulus and strength of hard coatings by relative method. Mater. Sci. Eng. A.

